# Analysis of chromatin accessibility in decidualizing human endometrial stromal cells

**DOI:** 10.1096/fj.201701098R

**Published:** 2018-01-08

**Authors:** Pavle Vrljicak, Emma S. Lucas, Lauren Lansdowne, Raffaella Lucciola, Joanne Muter, Nigel P. Dyer, Jan J. Brosens, Sascha Ott

**Affiliations:** * Tommy’s National Centre for Miscarriage Research, Warwick Medical School, University Hospitals Coventry and Warwickshire National Health Service (NHS) Trust, United Kingdom;; † Division of Biomedical Sciences, Warwick Medical School, University of Warwick, Coventry, United Kingdom;; ‡ Department of Computer Science, University of Warwick, Coventry, United Kingdom

**Keywords:** endometrium, decidualization, gene regulation, open chromatin, transposable elements

## Abstract

Spontaneous decidualization of the endometrium in response to progesterone signaling is confined to menstruating species, including humans and other higher primates. During this process, endometrial stromal cells (EnSCs) differentiate into specialized decidual cells that control embryo implantation. We subjected undifferentiated and decidualizing human EnSCs to an assay for transposase accessible chromatin with sequencing (ATAC-seq) to map the underlying chromatin changes. A total of 185,084 open DNA loci were mapped accurately in EnSCs. Altered chromatin accessibility upon decidualization was strongly associated with differential gene expression. Analysis of 1533 opening and closing chromatin regions revealed over-representation of DNA binding motifs for known decidual transcription factors (TFs) and identified putative new regulators. ATAC-seq footprint analysis provided evidence of TF binding at specific motifs. One of the largest footprints involved the most enriched motif—basic leucine zipper—as part of a triple motif that also comprised the estrogen receptor and Pax domain binding sites. Without exception, triple motifs were located within *Alu* elements, which suggests a role for this primate-specific transposable element (TE) in the evolution of decidual genes. Although other TEs were generally under-represented in open chromatin of undifferentiated EnSCs, several classes contributed to the regulatory DNA landscape that underpins decidual gene expression.—Vrljicak, P., Lucas, E. S., Lansdowne, L., Lucciola, R., Muter, J., Dyer, N. P., Brosens, J. J., Ott, S. Analysis of chromatin accessibility in decidualizing human endometrial stromal cells.

## INTRODUCTION

Decidualization of the endometrium occurs in all mammals in which the implanting embryo breaches the luminal endometrial epithelium and the trophoblast invades the maternal tissues (1). This process—defined by the transformation of endometrial stromal cells (EnSCs) into epithelioid decidual cells—occurs in concert with the secretory transformation of uterine glands, vascular remodeling, and influx of specialist immune cells, especially uterine NK cells (2). In pregnancy, decidual cells form a protective and nutritive matrix around the early conceptus that enables controlled trophoblast invasion and confers maternal immune tolerance of the antigenically distinct fetus (3–5).

In most mammals, decidualization is triggered by the implanting embryo; however, in a few species—that is, higher primates, 4 species of bats, the elephant shrew, and the common (Cairo) spiny mouse—decidualization is spontaneous, meaning that it is initiated during the midluteal phase of each cycle, independently of an implanting embryo (6, 7). Sustained progesterone signaling is essential to maintain the decidual phenotype of differentiated EnSCs (8). In the absence of pregnancy, falling ovarian progesterone production triggers a cascade of inflammatory events in the decidualizing endometrium that, upon recruitment and activation of leukocytes, becomes irrevocable and leads to partial tissue destruction, bleeding, and menstrual shedding (9). Recent studies have shown that the inextricable coupling of cyclic decidualization to menstruation and tissue repair bequeaths the uterus with additional reproductive traits, including the ability to reject invasive, but developmentally impaired embryos and to adapt to reproductive failure (10, 11).

Primary human EnSCs readily decidualize in culture in response to cAMP and progestin signaling in a manner that recapitulates closely the *in vivo* situation (12, 13). This model system has been used extensively to characterize the signaling pathways, downstream transcription factors (TFs), histone and DNA modifications, and gene networks that regulate this differentiation process [reviewed in detail in Gellersen *et al*. (2)]. Furthermore, DNase-seq has been used to map open chromatin regions, indicative of regulatory DNA, in decidualized EnSCs (14), but the dynamic changes in the chromatin landscape that underpin the transition of undifferentiated EnSCs to specialized decidual cells have not yet been characterized.

In this study, we used an assay for transposase accessible chromatin with sequencing (ATAC-seq) to profile the chromatin landscapes of primary human EnSCs in their undifferentiated state and upon decidualization. ATAC-seq utilizes the highly active transposase, Tn5, to interrogate the accessibility of the genome and map open chromatin regions. These putative *cis*-regulatory DNA regions can be additionally explored for footprints of TF binding (15). Footprints are small regions of tens of base pairs that are relatively resistant to transposase activity inside an otherwise highly accessible locus. We have generated a comprehensive map of chromatin regions that change dynamically upon decidualization, determined the TF binding motifs that are most enriched upon the opening or closing of chromatin, and interrogated footprints that are indicative of TF binding to *cis*-regulatory sequences in decidualizing EnSCs. Analysis of a conspicuous footprint that is indicative of the binding of a large protein complex led to the discovery of an abundant triple motif that consists of estrogen receptor (ESR), basic leucine zipper (bZIP), and Pax domain binding sequences in *Alu* elements, the most abundant transposable elements (TEs) in the human genome. We explored whether specific triple motifs could have been co-opted to regulate decidual gene expression in menstruating primates and examined the involvement of other TEs in the *cis*-regulatory landscape of differentiating human EnSCs.

## MATERIALS AND METHODS

### Ethical approval and sample collection

The study was approved by the National Health Service (NHS)National Research Ethics-Hammersmith and Queen Charlotte’s and Chelsea Research Ethics Committee (1997/5065). Endometrial biopsies were obtained from women who attended the Implantation Clinic, a dedicated research clinic at University Hospitals Coventry and Warwickshire National Health Service Trust. Timed midsecretory-phase biopsies were obtained using a Wallach Endocell endometrial sampler with written informed consent in accordance with the Declaration of Helsinki. ATAC-seq analysis was performed on undifferentiated and decidualizing EnSC cultures that were established from 1 nulliparous patient awaiting *in vitro* fertilization treatment and 2 recurrent miscarriage patients. Biopsies were obtained between 5 and 10 d after LH surge. None of the patients received hormonal therapy for at least 2 cycles before the biopsy cycle.

### Primary culture

Endometrial biopsies were collected in DMEM-F12 media that was supplemented with 10% dextran-coated charcoal (DCC)–stripped FBS and processed for primary EnSC culture as previously described (16). For decidualization studies, confluent monolayers of human EnSCs were incubated overnight at 37°C with 5% CO_2_ in phenol red–free DMEM/F-12 that contained 2% DCC with antibiotic/antimycotic and l-glutamine (2% medium). To induce differentiation, cells were treated in 2% DCC with 0.5 mM 8-bromoadenosine-cAMP (Sigma-Aldrich, Poole, United Kingdom) and 1 μM medroxyprogesterone acetate (MPA; Sigma-Aldrich) for the indicated time points. Control cultures were incubated in media that was supplemented with 2% DCC. Primary cultures were subjected to ATAC-seq at passage 2.

### ATAC-seq libraries

ATAC-seq was performed as previously described (15, 17), with some modifications. In brief, EnSCs (*n* = 3) were grown in 35-mm diameter tissue culture dishes. Confluent monolayers were washed with cold Dulbecco’s PBS, then lysed using ice-cold EZ lysis buffer (Sigma-Aldrich). Cells were scraped, then transferred to chilled prelabeled nuclease-free 1.5-ml microcentrifuge tubes. Samples were vortexed, left on ice for 5 min, then pelleted in a fixed-angle refrigerated benchtop centrifuge. The supernatant was carefully removed, and the pellet was washed once in EZ lysis buffer. The nuclear pellet was resuspended in the transposase reaction mix that contained 25 μl Tagment DNA buffer, 5 μl Tagment DNA enzyme, and 20 μl nuclease-free water (Nextera DNA Sample Preparation Kit; Illumina, Cambridge, United Kingdom). The transposition reaction was carried out for either 45 or 90 min at 37°C. Samples were purified using a Zymo DNA Clean and Concentrator-5 Purification Kit according to manufacturer instructions. In brief, DNA-binding buffer was added to the 50-μl sample that was then mixed and transferred to the column. Samples were centrifuged at 17,000 *g* for 30 s at room temperature. Flow-through was discarded, 200 μl DNA wash buffer added, and columns were centrifuged as described above. The wash and centrifugation steps were repeated twice. After removing the residual liquid, 23 μl prewarmed elution buffer was added to the columns. Samples were incubated for 2 min at room temperature, then centrifuged for 2 min to elute DNA. After purification, a 20 μl sample was added to a 0.2-ml PCR tube that contained the following reagents (Nextera DNA Sample Preparation Kit and Nextera Index Kit; Illumina): 5 μl index 1, 5 μl index 2, 15 μl Master mix, and 5 μl Primer Cocktail. Amplification was performed in a Veriti 96-Well Thermal Cycler (Applied Biosystems, Foster City, CA, USA) using the following PCR conditions: 72°C for 3 min, 98°C for 30 s, then 15 cycles of 98°C for 10 s, 63°C for 30 s, and 72°C for 1 min. Libraries were purified using AMPure XP beads according to the Illumina Nextera Kit recommended protocol. Amplified libraries were quantified using Qubit HS DNA Assay on a Qubit 2.0 Fluorometer (Thermo Fisher Scientific, Paisley, United Kingdom) according to the manufacturer’s instructions. Library sizes were assessed on an Agilent Technologies 2100 Bioanalyzer using the High Sensitivity DNA chip (Agilent Technologies, Santa Clara, CA, USA).

### Bioinformatic analysis

ATAC-seq libraries were sequenced with 100-bp paired ends on a HiSeq 2500 (Illumina) and mapped to the hg19 human genome assembly using bowtie2-2.2.6 (18) and samtools-1.2.0 (19). Peaks were called on a merged mapped file using MACS-2.1.0 (20), which led to the identification of 202,169 peaks. Of these peaks, 185,087 were deemed high confidence (*q* < 1 × 10^−4^) and were used for additional analysis. To determine differential chromatin opening, we used HTSeq-0.6.1 (21) to assign reads to peaks, and DESeq2 (22) was used to identify differential peaks. Peaks were then ranked according to their *P* value, taking into account the direction of the fold change, such that lower-rank values represent peaks that open upon decidualization, whereas high-rank values represent closing regions of chromatin. Data have been submitted to Gene Expression Omnibus (GEO; accession number: GSE104720).

### Comparison of peak data with other data sets

ATAC-seq data were compared with other available chromatin binding and accessibility data sets using BEDTools (23). Chromatin immunoprecipitation followed by sequencing (ChIP-seq) data sets from decidualizing stromal cells were obtained from the GEO for the following TFs: progesterone receptor (PGR; GSM1703567), Fos-like antigen (FOSL2; GSM1703568), and forkhead box O1 (FOXO1; GSM1703607). Formaldehyde-assisted isolation of regulatory elements with sequencing data for whole endometrium and DNaseI hypersensitivity data for decidualized EnSCs were obtained from GEO accession numbers GSM1011119 and GSE61793, respectively, whereas the Encyclopedia of DNA Elements (ENCODE; Stanford University, Stanford, CA, USA) DNaseI hypersensitivity data for 125 human cell and tissue types were obtained from the University of California, Santa Cruz (UCSC; Santa Cruz, CA, USA) genome browser track (24–26).

### Differential expression of proximal genes

ENCODE DNaseI hypersensitivity data were used to associate open chromatin regions and gene promoters with high confidence (25). A distal open chromatin region was deemed to be associated with a gene promoter if it was shown to physically interact and the distance was no more than 10 kb. Gene expression profiles of undifferentiated and decidualizing EnSCs were obtained by performing RNA-seq analysis on 3 independent samples (GEO accession number: GSE104721).

### Motif discovery and identification

Differentially opening chromatin regions were interrogated for enriched short-sequence motifs using HOMER v.4.8 (27). Background sets were generated from random genomic sequences of selected size near the transcription start site (TSS) of genes (± 50 kb), matching the GC/CpG distribution of the input. To additionally investigate the context of informative motifs, 50-bp sequences that surround enriched motifs were searched with Multiple Em for Motif Elicitation (MEME) and compared with motif databases using TomTom (28). Sequence conservation at each nucleotide position was calculated using WebLogo v.3 (29). Find Individual Motif Occurrences (FIMO) was used to identify instances of the triple motif sequence across the whole human genome (30). These locations were intersected with the RepeatMasker database retrieved *via* the UCSC Table Browser. Average ATAC-seq profiles and footprinting analysis were performed with Wellington (31).

### Conservation of *Alu* elements in primate genomes

To examine *Alu* conservation, *Alu* elements in the human genome were mapped to other primate species using blastn (32). We examined for conservation a total of 7120 *Alu* elements of >280 bp in length and overlapping gene-linked ATAC-seq peaks. Nucleotide sequences of 100 bp in length that spanned the 5′ and 3′ ends and surrounding sequences were mapped to other primate genomes. *Alu* loci for which orthologous loci in other primates that could not be established with confidence were excluded from the analysis. For remaining loci, the presence or absence of specific *Alu* elements at the orthologous loci was computed (see examples in Supplemental Fig. 7).

## RESULTS

### Altered chromatin accessibility upon decidualization

**Figure 1.**
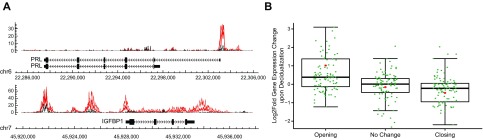
ATAC-seq analysis of decidualizing EnSCs. *A*) Dynamic chromatin changes at key decidual marker genes in differentiating EnSCs. ATAC-seq signals in undifferentiated and decidualizing cells are shown in black and red, respectively. Proximal promoters of *PRL* (prolactin; upper panel) and *IGFBP1* (IGF binding protein-1; lower panel) exhibit chromatin opening upon decidualization. *IGFBP1* has additional regions of opening chromatin that are upstream of the TSS and downstream of the termination site. *B*) Dynamic chromatin changes in decidualizing EnSCs correlate with differential gene expression. Box plots show differential expression of 100 genes nearby (within 10 kb of TSS) the most opening or closing regions. Log2 fold change in expression is shown on the *y* axis: +ve and −ve values represent genes that are up- and down-regulated, respectively. ATAC-seq peaks were grouped according to whether they open, close, or remain unchanged upon decidualization. Red asterisk shows mean log_2_-fold change.

To map dynamic chromatin changes upon decidualization, nuclei that were isolated from EnSC cultures, either untreated or decidualized with 8-bromoadenosine-cAMP and MPA for 4 d, were processed for ATAC-seq. This analysis accurately mapped the open chromatin regions in both cellular states. For example, expression of prolactin (*PRL*), a major decidual marker gene, is initiated in the endometrium at an alternative start site located at an additional noncoding exon, exon 1a, ∼6-kb upstream of the pituitary-specific TSS (33). As shown in **Fig. 1*A*** (upper panel), ATAC-seq analysis revealed selective chromatin opening immediately upstream of the nonpituitary *PRL* TSS in response to treatment with 8-bromoadenosine-cAMP and MPA, which confirmed that cells responded to deciduogenic signals. Another cardinal decidual marker gene is *IGFBP1* (IGF binding protein-1), and, again, chromatin opening across the proximal promoter region was apparent in differentiating EnSCs (Fig. 1*A*, lower panel). Additional regions of chromatin opening at the *IGFBP1* locus were mapped upstream of the TSS and downstream of the termination site.

The density of mapped ATAC-seq peaks as a function of genomic position provides a quantitative measure of chromatin accessibility. A total of 185,084 regions of accessible chromatin were mapped in EnSCs and ranked by the extent of opening or closing upon decidualization (Supplemental Table 1). On the basis of a stringent criterion (Bonferroni adjusted *P* < 0.05), 1225 and 278 loci opened or closed upon decidualization, respectively. The majority of these loci (55%) fell into gene introns, and 27% were found within 10 kb upstream of TSSs. Next, we examined the overlap in accessible chromatin regions that were identified by ATAC-seq and existing chromatin profiling data obtained either by DNaseI-seq analysis of decidualized EnSCs or by formaldehyde-assisted isolation of regulatory elements with sequencing analysis of whole endometrial tissue (14). More than 80% of open chromatin regions that were identified in this study were not mapped previously in either cultured decidual cells or whole endometrium (Supplemental Fig. 1); however, >95% of the open chromatin regions in EnSCs overlapped with DNaseI hypersensitive sites that were identified by DNaseI-seq analysis of 125 human cell and tissue types in the ENCODE project (25), which indicates that our analysis captured *bona fide* regulatory DNA regions.

The gain or loss of ATAC-seq peaks upon decidualization indicates altered TF binding that drives differential gene expression; however, both activating and repressive TFs can potentially bind at different regulatory sites, which renders it challenging to confidently predict gene expression from dynamic chromatin changes at specific loci alone. Nevertheless, analysis of 100 genes that are associated with either the most induced or repressed ATAC-seq peaks—within 10 kb of the promoter—revealed a strong, though not fully predictive, association with increased or decreased expression upon decidualization (*P* = 2.65 × 10^−9^, Student’s *t* test; Fig. 1*B*).

### Motif discovery and footprint analysis

**Figure 2.**
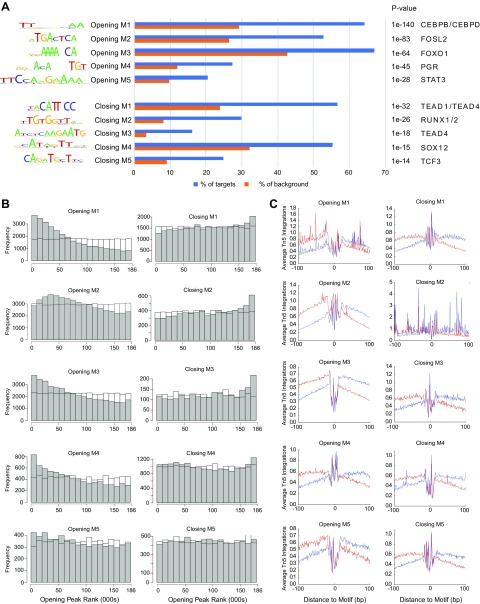
Gain and loss of TF binding motifs in dynamic chromatin regions. *A*) Top 5 most enriched TF binding motifs in opening and closing chromatin regions (see Supplemental Fig. 2 for full lists). The frequency (%) of opening or closing peaks featuring a given motif is shown relative to randomly selected genomic regions (±50 kb from TSSs, matching size, and GC/CpG content). On the basis of expression data and motif specificity, the most plausible TF for each motif is shown. *B*) The frequency of the 10 motifs was also determined across 185,084 open chromatin peaks in EnSCs. The *y* axis indicates the frequency of each motif across bins of 10,000 ATAC-seq peaks that are ranked on the *x* axis from the most opening (1) to the most closing peak (185,084). Nonshaded bars indicate expected frequency on the basis of genomic background frequency. *C*) Footprint analysis. Average ATAC-seq signals were calculated within 200-bp windows that were centered on enriched motifs in opening and closing ATAC-seq peaks. Red and blue indicate positive and negative strand cuts, respectively. A deep notch in the aggregated ATAC-seq signal at the motif, together with increased positive and negative strand reads at the 5′ and 3′ flanking regions, respectively, indicates occupancy by a DNA-binding factor. Footprints were present at all motifs, with the exception of closing motif 1 (M1). CEBP, CCAAT/enhancer binding protein; STAT3, signal transducer and activator of transcription; RUNX, runt-related transcription factor; SOX, SRY-box 12; TCF, transcription factor 3; TEAD, TEA domain transcription factor.

To gain insight into TFs that govern decidual gene expression, we performed *de novo* short-sequence motif enrichment analysis on the 1255 opening and 278 closing chromatin regions. This annotation revealed 17 significantly over-represented motifs in opening peaks (designated opening motifs 1–17) and 7 over-represented motifs in closing motifs 1–7 (**Fig. 2*A***, and Supplemental Fig. 2, and Supplemental Table 2). Of note, these motifs were associated strongly with the opening or closing of chromatin, not only in the subset of peaks used for motif discovery, but over the full set of peaks (Fig. 2*B* and Supplemental Fig. 3).

Binding of TFs to open chromatin should result in an ATAC-seq footprint. To examine the occupancy of informative binding sites, we calculated the average ATAC-seq signal centered on each motif (Fig. 2*C* and Supplemental Fig. 4). At the center of these plots, conserved motif sequences are reflected by spikes that result from the sequence-specific cutting bias of the Tn5 transposase. TF occupancy of a motif protects the DNA from transposase activity, which causes relative enrichment of reads across the binding site. This gives rise to the conspicuous pattern of excess positive and negative strand reads at 5′ and 3′ flanking regions of the motif, respectively. Footprint analysis indicated TF binding to *cis*-regulatory elements in all enriched motifs, except for opening motif 10 and closing motif 2. Next, we matched the motifs against known TF binding databases and examined the gene expression of the putative binding TFs in decidualizing EnSCs (Supplemental Fig. 5 and Supplemental Table 3). CCAAT/enhancer binding protein β and δ, FOSL2 (or FRA2), FOXO1, PGR, and signal transducer and activator of transcription 3 and 5 are essential TFs for decidualization (34–37), whereas TEA domain transcription factor 1 has been shown to repress decidualization (38). Of note, our top motifs in enriched ATAC-seq peaks corresponded to high-affinity binding sites for key decidual TFs (Fig. 2*A*). Furthermore, the most enriched motif in closing regions binds TEA domain transcription factor 1. We also compared ATAC-seq regions with existing ChIP-seq binding data for FOXO1 (GSM1703607), PGR (GSM1703567), and FOSL2 (GSM1703568) in differentiating EnSCs (39). FOXO1 and PGR binding was enriched in regions of opening chromatin (*P* < 1 × 10^−4^ and *P* < 1 × 10^−15^, respectively; Fisher’s exact test for 1000 opening and closing peaks), which is consistent with their role as core decidual TFs (Supplemental Fig. 6). Of interest, the presence of both PGR and FOSL2 binding sites within a chromatin region correlated with chromatin opening upon decidualization, whereas the presence of FOSL2 in the absence of PGR binding correlated with chromatin closure.

Next, we mined our RNA-seq data to identify novel decidual TFs that may bind other motifs in opening chromatin. This exercise yielded several candidate TFs that were not yet implicated in the promotion of decidualization, including RAR-related orphan receptor A, aryl hydrocarbon receptor nuclear translocator like, and Meis homeobox 1. Conversely, down-regulation of runt-related transcription factor 1 and 2, SRY-box 12, transcription factor 3, and ETS Proto-Oncogene 1 upon decidualization may account for the loss of chromatin accessibility in differentiating EnSCs. Taken together, our ATAC-seq data capture known modulators of decidualization, identify their putative target loci, and suggest novel TFs.

### Triple motif and *Alu* repeats

**Figure 3.**
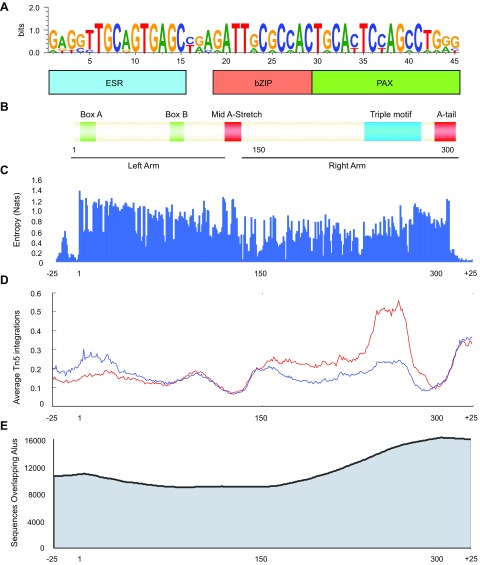
Triple motif in *Alu* elements. *A*) Short-sequence motif-finding analysis on 50-bp windows across opening motif 1 revealed a triple motif that consisted of ESR1, bZIP and Pax-like domain binding sequences. *B*) Triple motif is part of the 3′-end of *Alu* repetitive elements. Every triple motif in the genome overlaps with the right arm of *Alu* repetitive elements. *C*) Conservation, measured as entropy, of full-length *Alu* sequences in the genome was analyzed together with flanking sequences. Higher entropy, denoted as natural units of information (Nats), indicates higher conservation. *D*) Aggregated ATAC-seq signals across *Alu* sequences of at least 300 bp in length, together with 50 bp of surrounding sequence. Red and blue indicate positive and negative strand cuts, respectively. ATAC-seq peaks are enriched over the triple motif region, but are depleted in A-rich regions. *E*) Frequency of overlap of individual positions within the *Alu* elements and open chromatin regions. The 3′ end of *Alu* elements more frequently coincides with open chromatin than does the 5′ end.

Footprints around bZIP motifs (*e.g.*, opening motif 1) were noticeably wider than around other motifs (Fig. 2*C*), which suggests binding of multimeric complexes at these sites. Additional short-sequence motif analysis on 50-bp windows around these putative TF binding sites in opening motif 1 resulted in the discovery of an over-represented triple motif that consisted of ESR, bZIP, and Pax domain binding sequences in close proximity (**Fig. 3*A***). This triple motif occurs 47,499 times in open chromatin regions. A genome-wide search revealed that triple motifs overlap invariably with the 3′ end of *Alu* repetitive element sequences (Fig. 3*B*). Dimeric *Alu* elements are unique to primates and are classified into subfamilies according to their relative ages (40, 41). Triple motifs were found in all subfamilies of *Alu* elements, including the oldest, *Alu J*. Whereas they share the same level of sequence conservation with other *Alu* regions (Fig. 3*C*), triple motifs were much more dynamically regulated upon decidualization (Fig. 3*D*). Indeed, the 3′ end of *Alu* elements overlaps ATAC-seq peaks more frequently than does the 5′ end (*P* = 2.2 × 10^−16^, binomial test; Fig. 3*E*).

**Figure 4.**
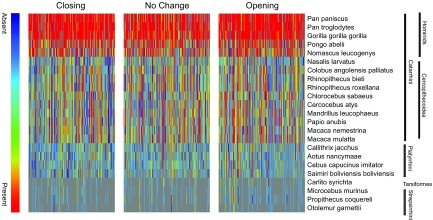
*Alu* conservation and co-option in primate species. Heat map shows the conservation of *Alu* elements across primate species. Only *Alus* that were associated with genes (within 10 kb of TSSs) were examined. Green indicates presence, red absence, and gray inconclusive. *Alu* conservation decreases with evolutionary distance.

Next, we identified 7120 *Alu* elements that overlap with open chromatin regions that are associated with genes and examined their conservation in menstruating primates. Menstruation is well documented in Catarrhines (humans, apes, and Old World monkeys), but is absent in Strepsirrhines (*e.g.*, bushbabies/galagos). The prevalence of menstruation in Platyrrhini (New World monkey) is much less clear, with conflicting observations reported for certain species (7, 42). As shown in **Fig. 4**, the level of conservation of informative *Alu* was high in Catarrhines, but not in Strepsirrhines. An intermediate level of conservation was observed in Platyrrhini, including squirrel monkey (Saimiri) and marmoset (Callithrix), 2 reportedly nonmenstruating primates. To confirm these findings, we examined a set of genes that were differentially expressed upon decidualization that are putatively regulated by *Alu* elements in opening or closing ATAC-seq peaks (Supplemental Table 4). Of interest, among these genes are several that encode key decidual TFs—for example, *CEBPD* and *WT1* (Wilms tumor 1)—and signal intermediates, such as *PROK1* (prokineticin 1) and *SGK1* (serum/glucocorticoid regulated kinase 1) (13, 43, 44). Taken together, these observations suggest that the triple motif in *Alu* elements contributes to the *cis*-regulatory landscape that governs the spontaneous decidualization and menstruation in the primate lineage; however, there was no evidence that *Alu* repeats in dynamic *vs*. nondynamic chromatin regions in decidualizing EnSCs were evolutionarily more conserved (Fig. 4).

### Contribution of other TEs

**Figure 5.**
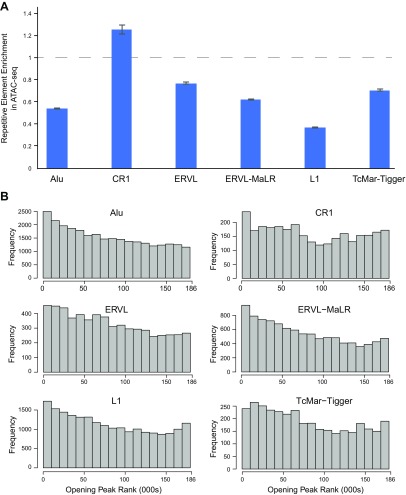
Involvement of TEs in dynamic chromatin changes upon decidualization. *A*) Relative frequency of ATAC-seq peaks overlapping repetitive elements identified by RepeatMasker. Of the six most abundant TE families in ATAC-seq peaks, only one [chicken repeat 1 (CR1)] exceeds the frequency that is expected by chance. *B*) The frequency of TE families was also determined across 180,000 open chromatin peaks. The *y* axis indicates the frequency of each family across bins of 10,000 peaks that are ranked on the *x* axis from the most opening (1) to the most closing peak (185,084).

To determine the involvement of other TEs in the open chromatin landscape of human EnSCs, we overlapped ATAC-seq data with repetitive elements that were identified by RepeatMasker. In 5 of 6 of the most abundant repetitive families, the frequency of overlap with ATAC-seq peaks was lower than expected by chance (*P* < 1 × 10^−15^, Fisher’s exact test). Only one family—chicken repeat 1 elements—demonstrated a higher frequency than expected (*P* < 1 × 10^−15^, Fisher’s exact test; **Fig. 5*A***). Next, we examined the contribution of TEs to the dynamic chromatin changes in decidualizing cells by examining their frequency in opening peaks. This analysis identified additional exapted TEs, including the Eutherian-specific hAT-Charlie DNA transposon that has been previously shown to confer cAMP and progesterone responsiveness in decidualizing EnSCs (45). Other notable TEs included Line1 and the exogenous retroviral-derived ERVL (Fig. 5*B* and Supplemental Fig. 8). Of interest, low complexity and simple repeats were more likely to close during decidualization (Supplemental Fig. 8). Finally, chromatin accessibility was predominantly unchanged upon decidualization in regions of Satellite and ERVK sequences. Thus, whereas repetitive elements are generally under-represented in open chromatin in EnSCs, they nevertheless function frequently as part of the regulatory landscape that renders EnSCs responsive to cAMP and progestin signaling.

## DISCUSSION

Decidualization of human EnSCs depends on the convergence of the cAMP and progesterone signaling pathways as well as the induction and/or activation of core decidual TFs (2, 12). Here, we present the first characterization of the changes in chromatin accessibility upon differentiation of human EnSCs into decidual cells. Opening ATAC-seq peaks result from the cooperative binding of TFs in place of a canonical nucleosome. Congruently, the most highly enriched *cis*-regulatory motifs upon decidualization represent high-affinity binding sites for core decidual TFs, including C/EBPs, FOXO1, signal transducers and activators of transcription, PGR, and members of the activator protein-1 family of transcription factors, most prominently FOSL2 (2, 34). Although multiple TFs can potentially bind a given motif, the mining of published ChIP-seq data confirms increased binding of both FOXO1 and PGR to opening chromatin regions in decidualizing cells (34, 39). Several studies have reported that cooperation between core decidual TFs, which is often mediated *via* direct protein-protein interaction, modifies the transcription output of individual TFs (46–48). A case in point is FOSL2, which has been identified as a putative PGR coregulator in a ChIP-seq analysis of the PGR, cistrome, in decidualizing EnSCs (34). Our data demonstrate that the presence of both PGR and FOSL2 binding sites within a DNA region correlates with increased chromatin accessibility upon decidualization, whereas sites with only FOSL2 motifs were more likely to close. We identified a total of 17 enriched motifs in opening chromatin, all of which, bar one (opening motif 10), demonstrated evidence of TF binding in ATAC-seq footprints. Cross-referencing of these binding motifs against TFs that are up-regulated at the mRNA level upon decidualization identified several putative novel decidual transcriptional regulators, including RAR-related orphan receptor A, aryl hydrocarbon receptor nuclear translocator like, and Meis homeobox 1. Reduced chromatin accessibility was equally associated with the loss of binding motifs for TFs that are repressed upon decidualization, most notably, runt-related transcription factor 1 and 2, SRY-box 12, transcription factor, and ETS Proto-Oncogene 1. Whereas gene expression was not fully predicted by chromatin changes at specific loci, overall, the changes in chromatin accessibility—leading to either the loss or enrichment of *cis*-regulatory DNA elements in both promoters and at distal sites—correlated strongly with differential gene expression in decidualizing cells.

There is compelling evidence that the emergence of decidual cells in Eutherian (placental) mammals was driven by large-scale integration of numerous TEs that contributed *cis*-regulatory elements that were coopted by EnSC-specific TFs to drive decidual gene expression (14). Most families of repetitive elements, including *Alu* and *L1* elements, are under-represented in the open chromatin landscape of EnSCs, which suggests active repression; however, several families of TEs were over-represented in opening chromatin upon decidualization, which supports the notion that they contributed essential functional DNA sequences that enabled the endometrium to accommodate an invasive placenta. Among Eutherian mammals, spontaneous decidualization coupled with menstruation is a rare phenomenon, with higher primates accounting for 93% of all known menstruating species (6, 7). A striking observation in this study was the discovery of a singular triple TF binding motif, embedded in primate-specific dimeric *Alu* elements, that was not only linked strongly to chromatin opening upon decidualization, but that also was conserved in menstruating but not in nonmenstruating primates. The precise nature of the TF complex that binds this triple motif, which consists of contiguous ESR, bZIP, and Pax domain binding sequences, requires additional investigation, although ESR sites in *Alu* sequences have been shown previously to be bound *in vivo* (49, 50). As estradiol is not part of our differentiation protocol, our findings potentially point to a role for the unliganded ESR in regulating decidual gene expression, a phenomenon that has already been described for PGR and androgen receptor (12, 51). Triple motifs in *Alu* elements are abundant throughout the genome, which indicates that the binding of a multimeric transcriptional complex upon decidualization is likely dependent on the local chromatin context. Whereas our observations are intriguing, it is possible that the emergence of *Alu* triple motif and menstruation in primates may not be functionally linked, but merely reflects the co-occurrence of two evolutionary events with similar timing.

Although spontaneous decidualization in primates and other species evolved independently, menstruating mammals share a number of reproductive features, including spontaneous ovulation, a deeply invading hemochorial placenta, and few well-developed offspring. The convergence of these reproductive traits suggests an adaptive value for species that possess them (7); however, the reproductive advantage of menstruation has been the subject of intense controversy (7, 52–54), especially in view of the health burden associated with abnormal menstruation and menstruation-associated disorders, such as endometriosis (2). Furthermore, overwhelming evidence indicates that aberrant decidualization causes a spectrum of clinical disorders, including implantation failure and recurrent pregnancy loss (11, 13, 55). By mapping the dynamic changes in the chromatin landscape in differentiating human EnSCs, our work constitutes a step toward the full elucidation of the regulatory networks that drive decidualization, which ultimately may contribute to the development of more targeted approaches for the treatment of reproductive failure.

## Supplementary Material

This article includes supplemental data. Please visit *http://www.fasebj.org* to obtain this information.

## Abbreviations

ATAC: assay for transposase accessible chromatin
bZIP: basic leucine zipper
ChIP-seq: chromatin immunoprecipitation followed by sequencing
DCC: dextran-coated charcoal
EnSC: human endometrial stromal cell
ESR: estrogen receptor
FOSL2: Fos-like antigen
FOXO1: forkhead box O1
GEO: Gene Expression Omnibus
MPA: medroxyprogesterone acetate
PGR: progesterone receptor
seq: sequencing
TE: transposable element
TF: transcription factor
TSS: transcription start site
