# Supplementary material for: Analysis of chromatin accessibility in decidualizing human endometrial stromal cells
Source: FASEB J. 2018 Jan 8;32(5):2467–77. doi: 10.1096/fj.201701098R (PMC6040682; doi:10.1096/fj.201701098R)
Supplement: Supplementary file 1 [file fj.201701098R.sf1.pdf]

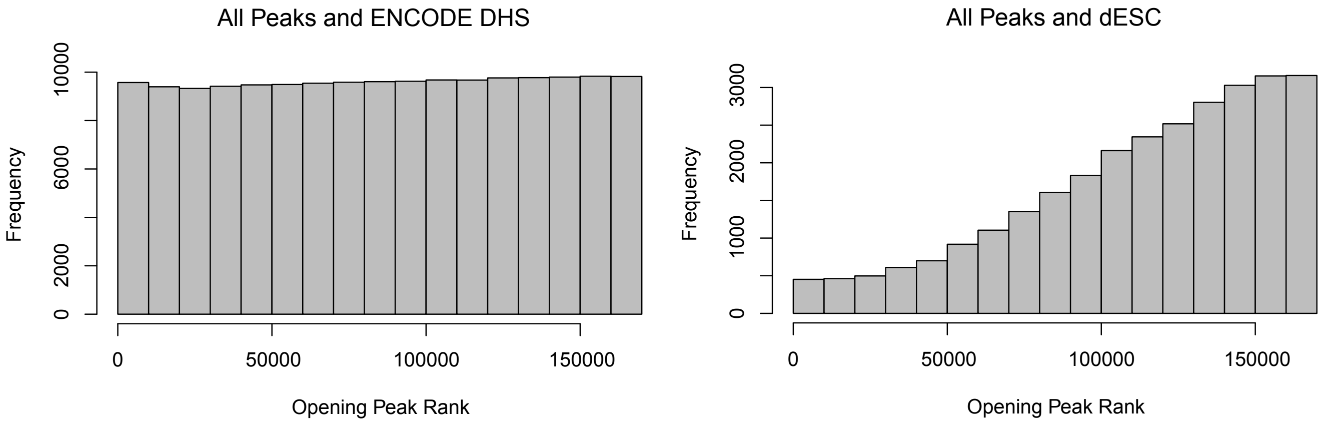

Figure S1. Previously published open chromatin regions are primarily overlapping with closing regions.

Open chromatin regions observed in our data were ranked from the most strongly opening regions down to the most strongly closing regions during decidualization. Regions were grouped into bins according to the ranking and numbers of regions overlapping with published datasets were determined for each bin. Regions found to overlap with ENCODE data from 125 cell lines are uniformly distributed across this ranking whereas open regions reported for decidualized EnSCs are preferentially closing.
