# Supplementary figures and images for: Analysis of chromatin accessibility in decidualizing human endometrial stromal cells
Source: FASEB J. 2018 Jan 8;32(5):2467–77. doi: 10.1096/fj.201701098R (PMC6040682; doi:10.1096/fj.201701098R)

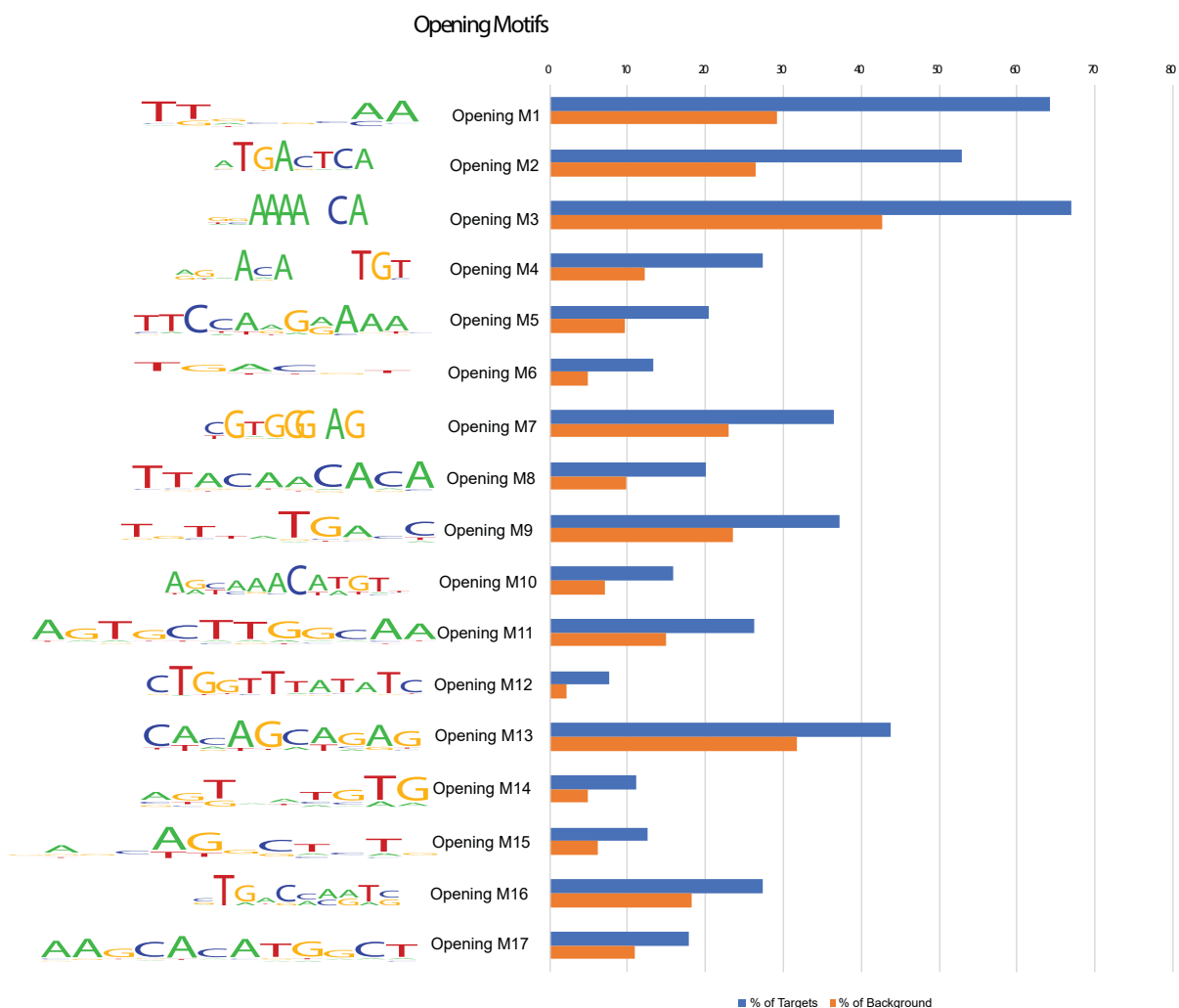

### Closing Motifs

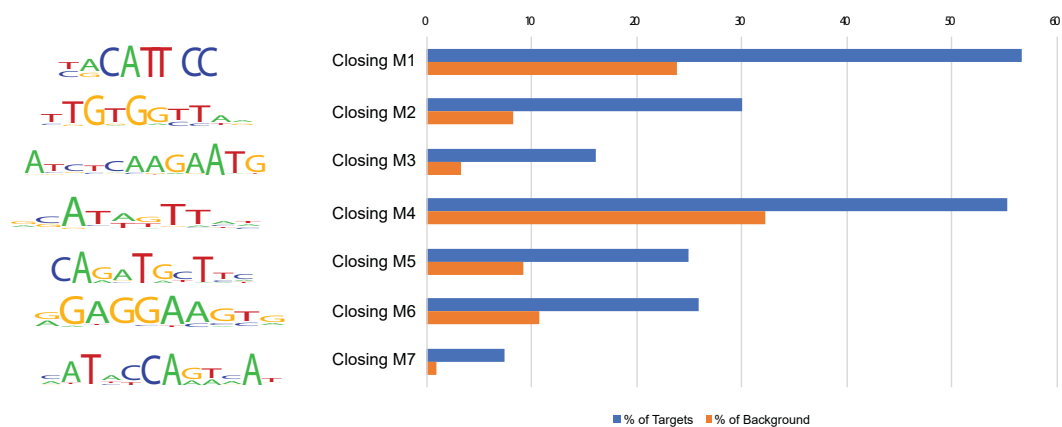

Figure S2. Full list of opening and closing motifs (expansion of Figure 2A)

Supplement: Supplementary file 2 [file fj.201701098R.sf2.pdf]

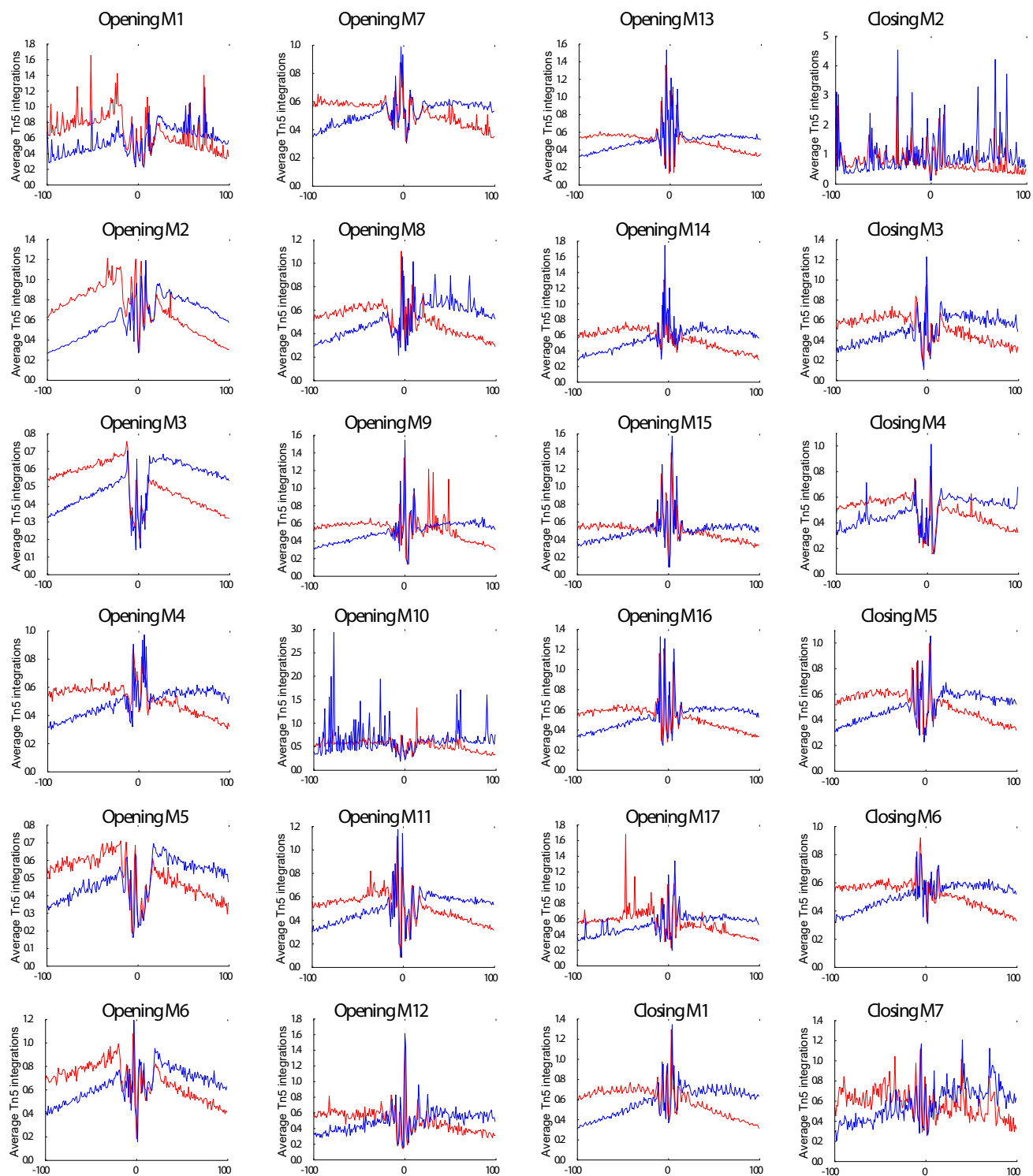

Figure S4. Average Tn5 integration patterns for all motifs (expansion of Figure 2C)

Supplement: Supplementary file 4 [file fj.201701098R.sf4.pdf]

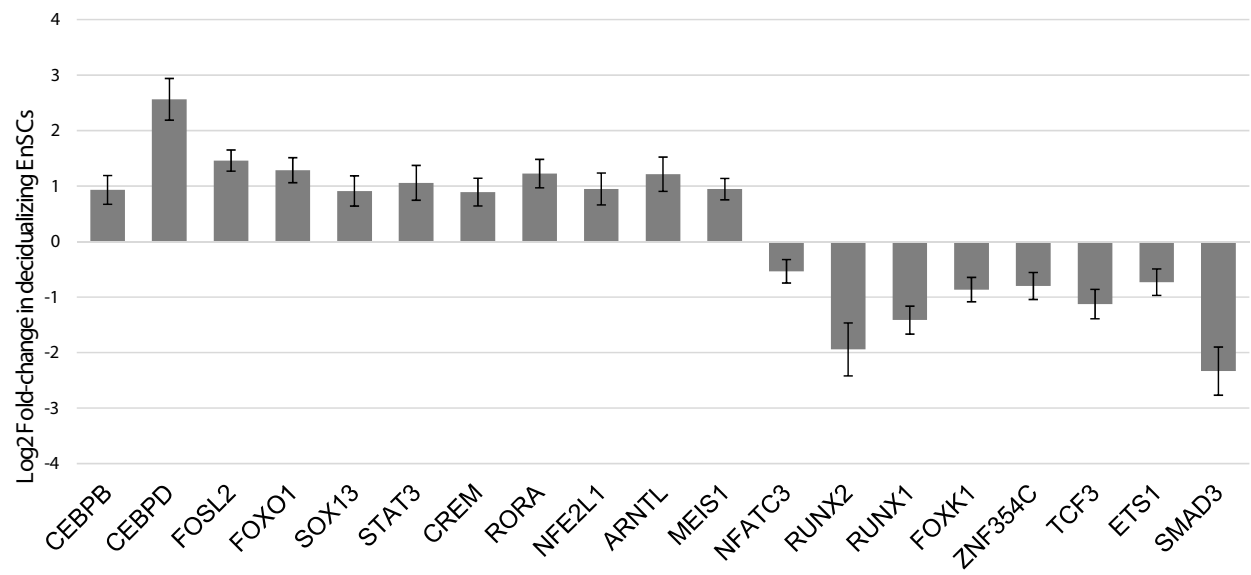

Figure S5. Expression of TFs in decidualizing EnSCs

Supplement: Supplementary file 5 [file fj.201701098R.sf5.pdf]
