# Supplementary material for: Analysis of chromatin accessibility in decidualizing human endometrial stromal cells
Source: FASEB J. 2018 Jan 8;32(5):2467–77. doi: 10.1096/fj.201701098R (PMC6040682; doi:10.1096/fj.201701098R)
Supplement: Supplementary file 3 [file fj.201701098R.sf3.pdf]

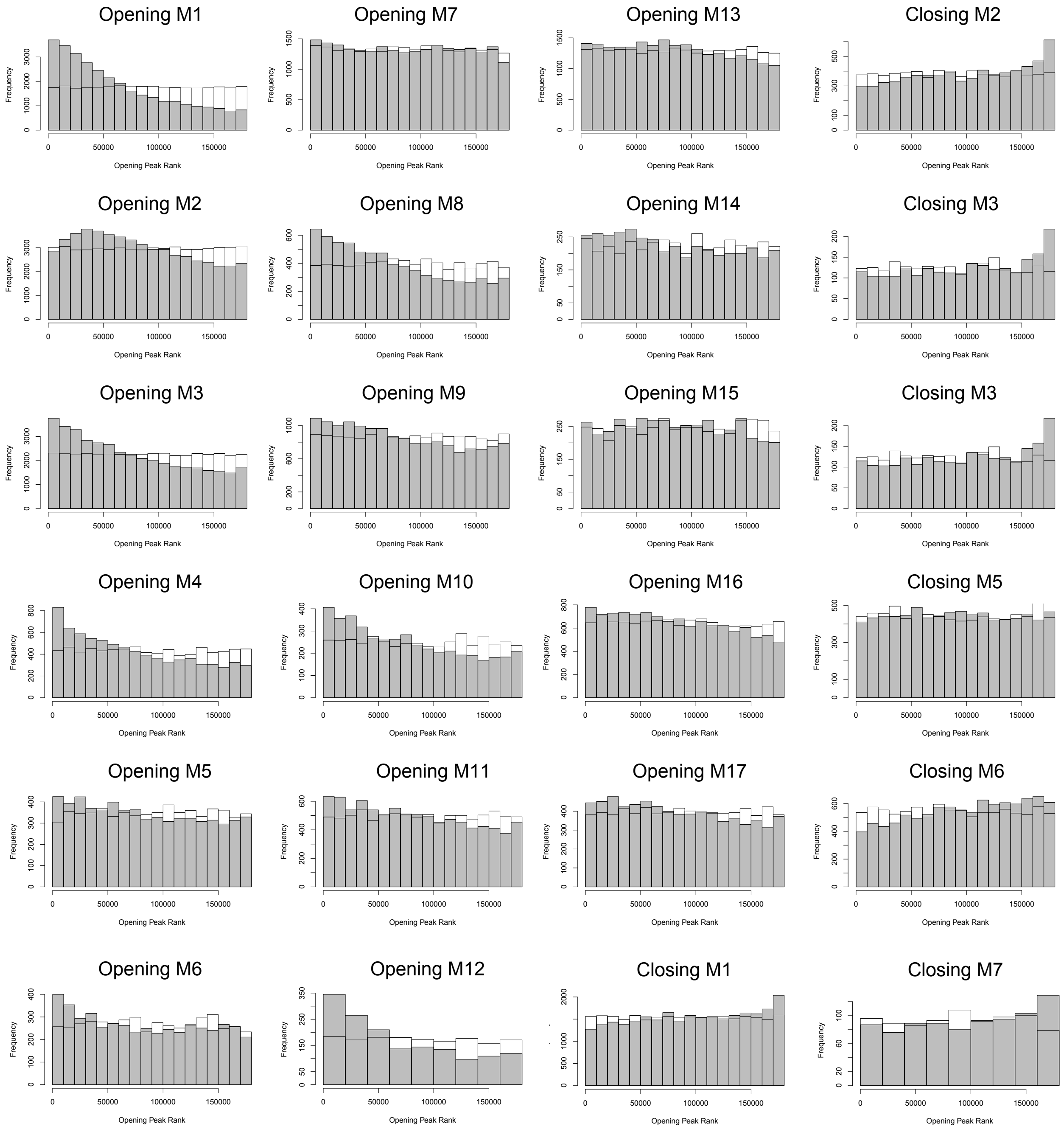

Figure S3. Association of motifs with chromatin opening or closing across entire ATAC-seq peak dataset for all motifs (expansion of Figure 2B)
