# Supplementary material for: Analysis of chromatin accessibility in decidualizing human endometrial stromal cells
Source: FASEB J. 2018 Jan 8;32(5):2467–77. doi: 10.1096/fj.201701098R (PMC6040682; doi:10.1096/fj.201701098R)
Supplement: Supplementary file 6 [file fj.201701098R.sf6.pdf]

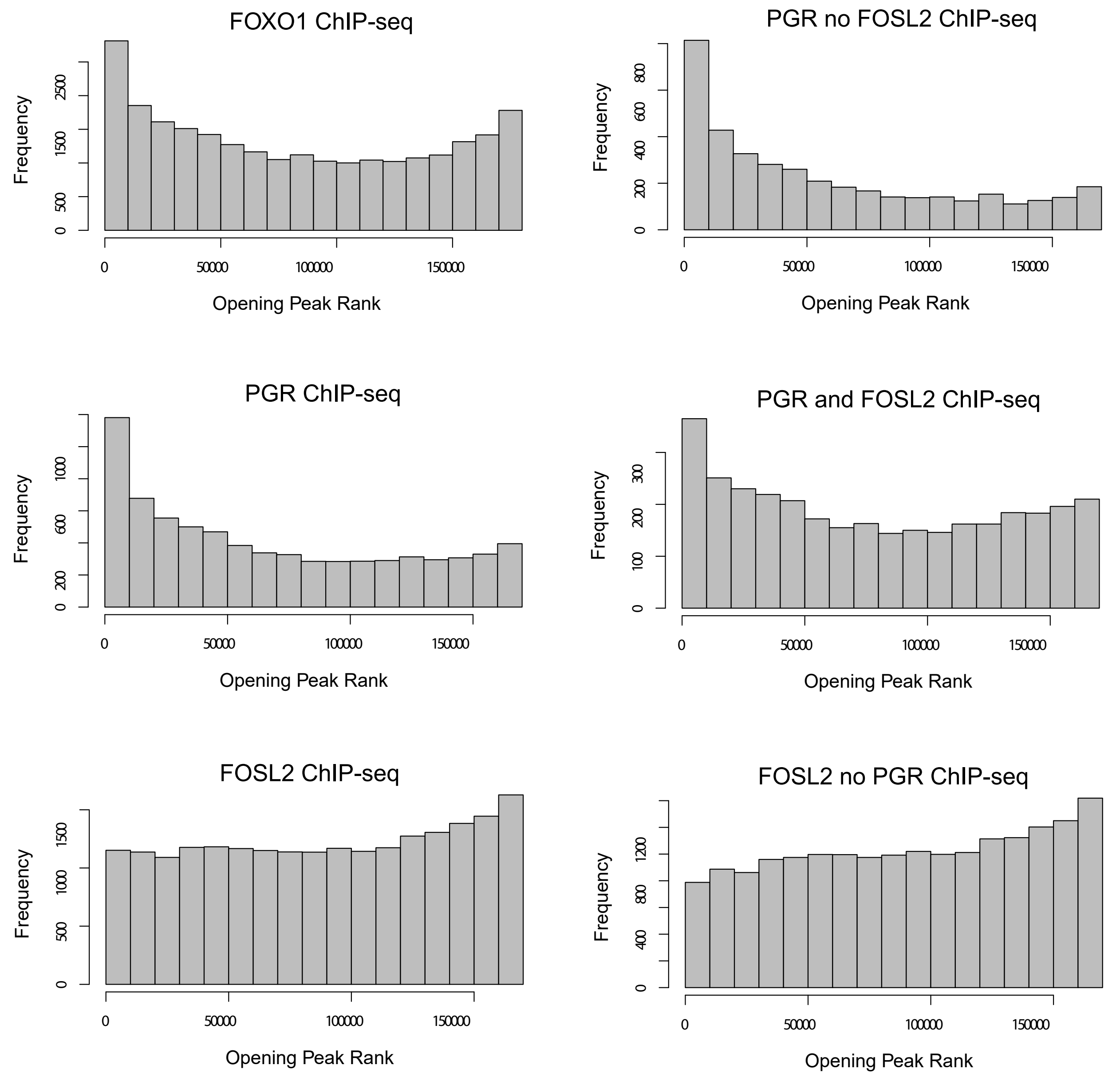

Figure S6. Mapping known binding sites of key TFs in decidualization to opening and closing chromatin regions.

Binding regions of FOXO1, PGR, and FOSL2 determined in published ChIP-seq datasets for EnSCs were mapped to open chromatin regions detected in this study. ATAC-seq peaks were ranked from most opening to most closing peaks. FOXO1 and PGR binding is enriched in opening chromatin regions. FOSL2 binding is associated with closing chromatin, while the subset of FOSL2-bound peaks that also bind PGR is associated with opening chromatin.
