# Supplementary material for: Analysis of chromatin accessibility in decidualizing human endometrial stromal cells
Source: FASEB J. 2018 Jan 8;32(5):2467–77. doi: 10.1096/fj.201701098R (PMC6040682; doi:10.1096/fj.201701098R)
Supplement: Supplementary file 7 [file fj.201701098R.sf7.pdf]

A

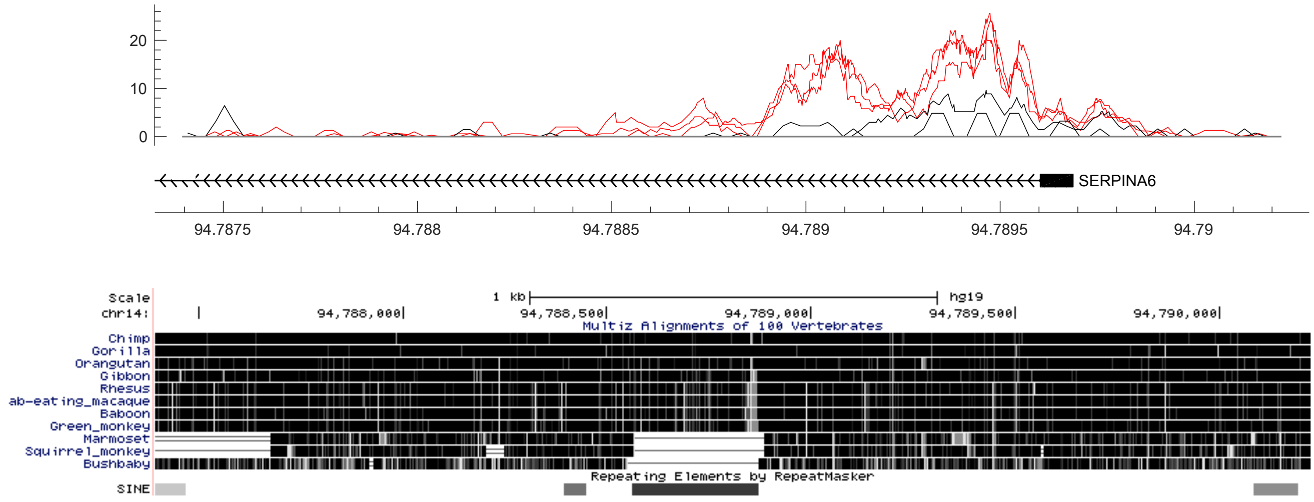

B

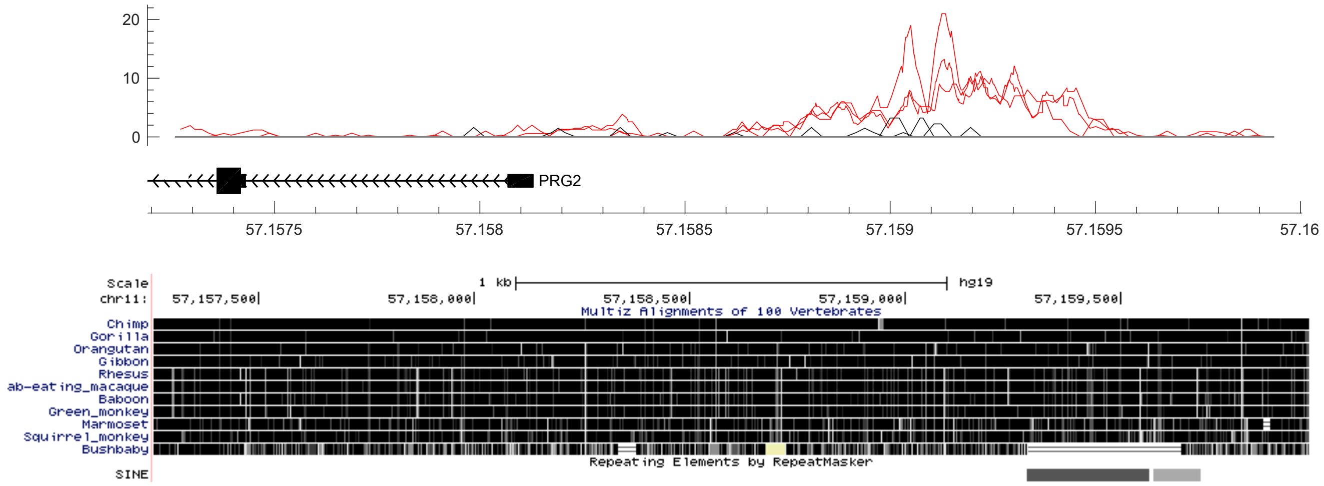

Figure S7. Examples of conserved Alu elements near decidualization genes.

Upper panel shows an example of an Alu element missing in marmoset, squirrel monkey and bushbaby. Alu element shown in lower panel is conserved up to squirrel monkey.
