# Supplementary material for: Analysis of chromatin accessibility in decidualizing human endometrial stromal cells
Source: FASEB J. 2018 Jan 8;32(5):2467–77. doi: 10.1096/fj.201701098R (PMC6040682; doi:10.1096/fj.201701098R)
Supplement: Supplementary file 8 [file fj.201701098R.sf8.pdf]

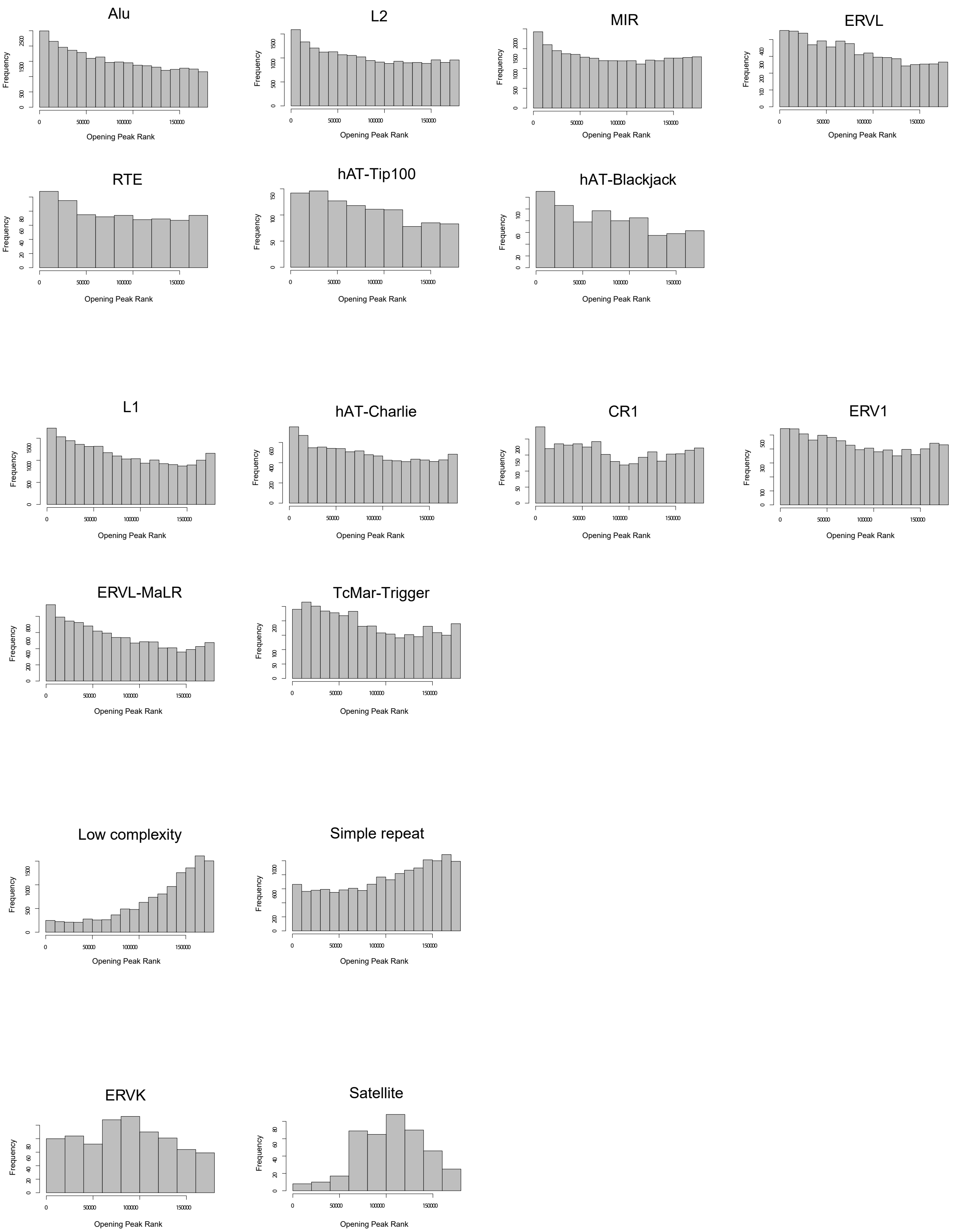

Figure S8. Association of other repetitive elements with opening and closing of chromatin (expansion of Figure 5B).
